# Chemotherapy-induced myasthenic crisis in thymoma treated with primary chemotherapy with curative intent on mechanical ventilation: a case report and review of the literature

**DOI:** 10.1186/s13256-020-02601-8

**Published:** 2021-02-02

**Authors:** Giorgio Patelli, Katia Bencardino, Federica Tosi, Mariateresa Pugliano, Francesca Lanzani, Alessandro Innocenti, Alessandro Rinaldo, Gianluca Mauri, Giulio Cerea, Andrea Sartore-Bianchi, Massimo Torre, Elio Clemente Agostoni, Salvatore Siena

**Affiliations:** 1 Niguarda Cancer Center, Struttura Complessa Oncologia Falck, Grande Ospedale Metropolitano Niguarda, Milan, Italy; 2 Department of Oncology and Hemato-Oncology, Università degli Studi di Milano, Milan, Italy; 3 Struttura Complessa Immunoematologia e Medicina Trasfusionale, Grande Ospedale Metropolitano Niguarda, Milan, Italy; 4 Struttura Complessa Neurologia and Stroke Unit, Grande Ospedale Metropolitano Niguarda, Milan, Italy; 5 De Gasperis Cardio-Thoracic Center, Struttura Complessa Chirurgia Toracica, Grande Ospedale Metropolitano Niguarda, Milan, Italy

**Keywords:** Thymoma, Myasthenia gravis, Myasthenic crisis, Chemotherapy, Plasma exchange, Case report

## Abstract

**Background:**

Thymoma is an uncommon cancer often associated with myasthenia gravis, an autoimmune disorder of the neuromuscular junction characterized by muscular fatigability. In patients with advanced nonmetastatic thymoma, primary chemotherapy may be required to induce tumor shrinkage and to achieve radical resection. Cancer chemotherapy has been anecdotally reported as a trigger factor for worsening of myasthenia gravis in thymic epithelial cancers. The study of uncommon cases of chemotherapy-related myasthenic crisis is warranted to gain knowledge of clinical situations requiring intensive care support in the case of life-threatening respiratory failure.

**Case presentation:**

We report a case of an 18-year-old Caucasian woman with advanced Masaoka-Koga stage III type B2 thymoma and myasthenia gravis on treatment with pyridostigmine, steroids and intravenous immunoglobulins, who developed a myasthenic crisis 2 hours after initiation of cyclophosphamide/doxorubicin/cisplatin primary chemotherapy. Because of severe acute respiratory failure, emergency tracheal intubation, mechanical ventilation, and temporary (2 hours) discontinuation of chemotherapy were needed. Considering the curative intent of the multimodal therapeutic program, we elected to resume primary chemotherapy administration while the patient remained on mechanical ventilation. After 24 hours, the recovery of adequate respiratory function allowed successful weaning from respiratory support, and no further adverse events occurred. After 3 weeks, upon plasma exchange initiation with amelioration of myasthenic symptoms, a second course of chemotherapy was given, and in week 6, having documented partial tumor remission, the patient underwent radical surgery (R0) and then consolidation radiation therapy with 50.4 Gy in 28 fractions in weeks 15–20.

**Conclusions:**

This case report, together with the only four available in a review of the literature, highlights that chemotherapy may carry the risk of myasthenic crisis in patients affected by thymoma and myasthenia gravis. To our knowledge, this is the first reported case of chemotherapy continuation on mechanical ventilation in a patient with chemotherapy-induced myasthenic crisis requiring tracheal intubation. The lesson learned from the present case is that, in selected cases of advanced thymoma, the paradoxical worsening of myasthenia gravis during chemotherapy should not be considered an absolute contraindication for the continuation of primary chemotherapy with curative intent.

## Background

Thymoma is an uncommon epithelial cancer associated in nearly half of cases with myasthenia gravis (MG), an autoimmune disorder of the neuromuscular junction characterized by muscular fatigability. In patients with advanced thymoma, primary chemotherapy is considered the standard of care, with the aim of achieving tumor shrinkage and facilitating radical surgery [1]. While several drugs are known to worsen MG, cancer chemotherapy has been anecdotally reported as a trigger event for myasthenic crisis (MC) in four case reports [2–6]. Here, we present a case of advanced thymoma treated with primary chemotherapy that caused an acute-onset MC requiring tracheal intubation and therapeutic plasma exchange (PEX), with relevant benefit from early resumption of anticancer treatment during mechanical ventilation.

## Case presentation

### Clinical presentation and diagnosis

**Fig. 1:**
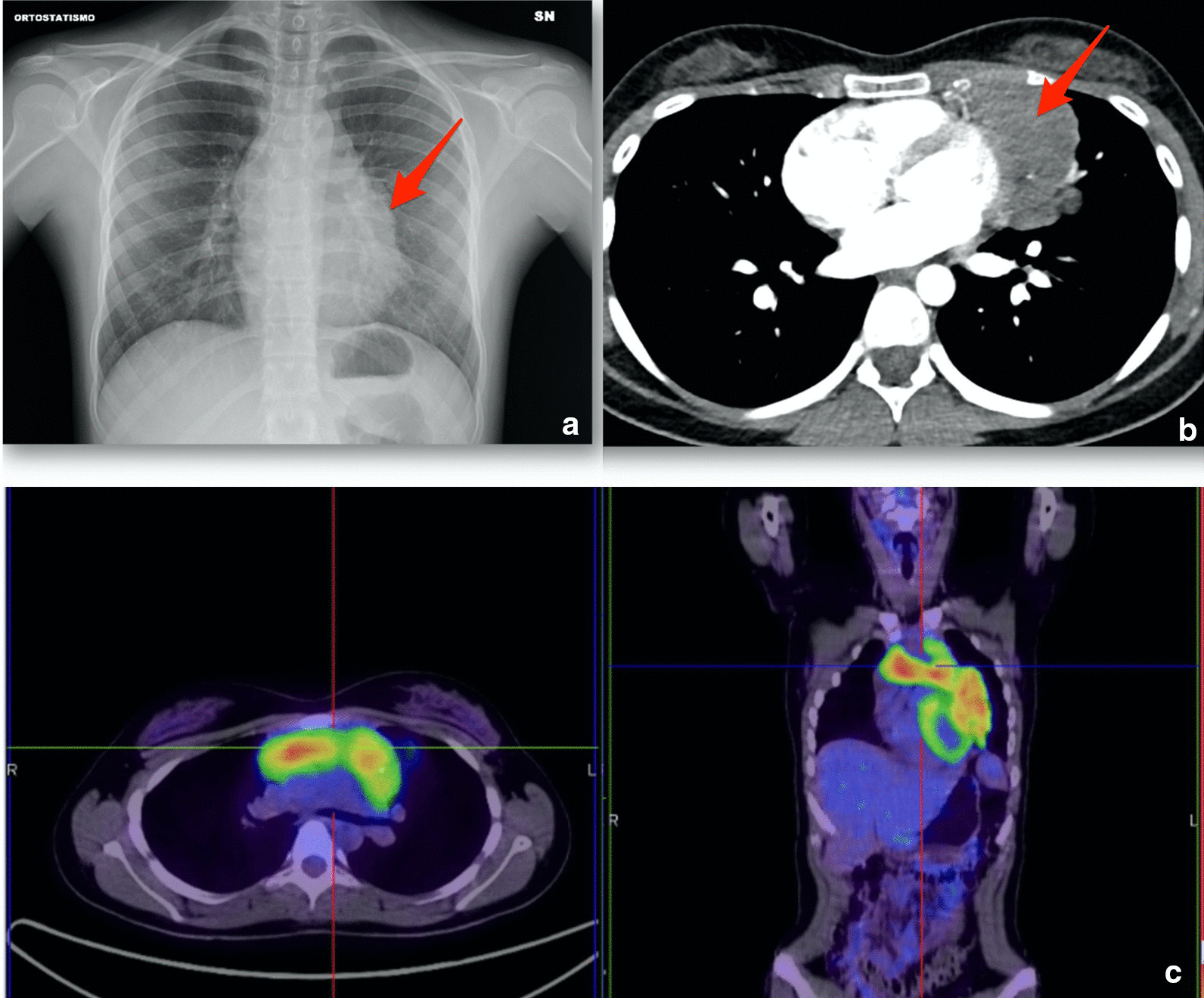
Patient imaging. **a** Left chest X-ray with enlarged mediastinum (see arrow). **b** Chest computed tomography scan showing the mediastinal mass (see arrow). **c** ^18^PET-FDG limited radiotracer uptake in mediastinum

An 18-year-old Caucasian woman with unremarkable medical history was admitted to the emergency room due to neurological symptoms. She presented with month-long worsening dysphagia, rhinolalia, bilateral ptosis, and skeletal muscle weakness. Signs of muscle fatigability were elicited at the physical exam and then confirmed by electromyography, consistently with neuromuscular junction disease. Blood tests were unremarkable with the exception of acetylcholine receptor antibodies (43 nmol/l, normal if < 0.5 nmol/l). Hence, MG class III, according to the Myasthenia Gravis Foundation of America (MGFA) scale, was diagnosed [7, 8]. Partial regression of symptoms to class II MG was achieved by oral administration of pyridostigmine 60 mg every 3 hours during the daytime, pyridostigmine sustained-release 180 mg orally before sleeping, prednisone 25 mg orally each day, and immunoglobulins 0.4 g/kg intravenously for 5 days [8, 9]. Chest computed tomography (CT) revealed a 12 cm anterior mediastinal mass suspicious for thymoma, with confirmed infiltration of the anterior chest wall, lung parenchyma, and pericardium at chest magnetic resonance imaging with contrast. CT-guided fine needle aspiration biopsy yielded a histological diagnosis of B2 thymoma [10]. Due to the young age, a family history of cancer was excluded. Physical exam was negative for signs of mediastinal syndrome and superficial lymph nodes. Metastatic disease was excluded by 18-fluorodeoxyglucose positron emission tomography (^18^FDG-PET). Patient imaging is shown in Fig. 1. Accordingly, the clinical stage was cT3 cN0 cM0 (Masaoka-Koga III) [11]. After multidisciplinary team discussion, the tumor was defined as unresectable, and the patient was offered primary chemotherapy with intravenous cyclophosphamide 500 mg/m^2^, doxorubicin 50 mg/m^2^ and cisplatin 50 mg/m^2^ (CAP regimen) on day 1 every 21 days [12]. Primary prophylaxis of chemotherapy-induced neutropenia was not planned due to the potential risk of worsening MG, without evidence about the safety of administering granulocyte colony-stimulating factor in unstable myasthenic patients [13]. Ovarian preservation by gonadotropin-releasing hormone analogue leuprorelin was preferred over oocyte retrieval to expedite the start of chemotherapy [14]. Standard pre-medication with intravenous administration of ondansetron 8 mg, dexamethasone 8 mg and saline was chosen [15].

### Chemotherapy-induced myasthenic crisis

Two hours after starting administration of the first course of chemotherapy, following doxorubicin bolus, the patient experienced sudden onset of acute dyspnea with severe respiratory failure (oxygen saturation 75% on room air), refractory to oxygen therapy and bolus hydrocortisone 500 mg and chlorphenamine 10 mg administered intravenously [16]. Physical exam was negative for bronchospasm with diminished vesicular breath sounds. Due to unstable clinical conditions and development of acute respiratory acidosis (pH 7.21, partial pressure of carbon dioxide 88 mmHg), tracheal intubation and mechanical ventilation in the intensive care unit (ICU) were needed. Urgent chest CT scan excluded acute cardiopulmonary events. Life-threatening MGFA class V MC was diagnosed, likely triggered by chemotherapy, thus classified as grade 4 according to the Common Terminology Criteria for Adverse Events version 5.0 (CTCAE v5.0) [8, 17]. Chemotherapy was held for 2 hours and then, considering its curative intent and in agreement with the family, it was resumed and fully administered during mechanical ventilation. After 24 hours, upon remission of MC and weaning from ventilation, the patient was extubated and could continue receiving MG treatment, together with starting PEX once every three days to control MG and prevent new crises (by replacing 1 plasma volume with 5% human albumin) [18]. In week 2, owing to CTCAE v5.0 grade 3 febrile neutropenia (absolute neutrophil count 60/mm^3^ and body temperature 38.4 °C), the patient was successfully treated with empirical piperacillin/tazobactam 4 g/0.5 g intravenously every 6 hours until recovery [19].

### Curative treatment and outcome

**Fig. 2:**
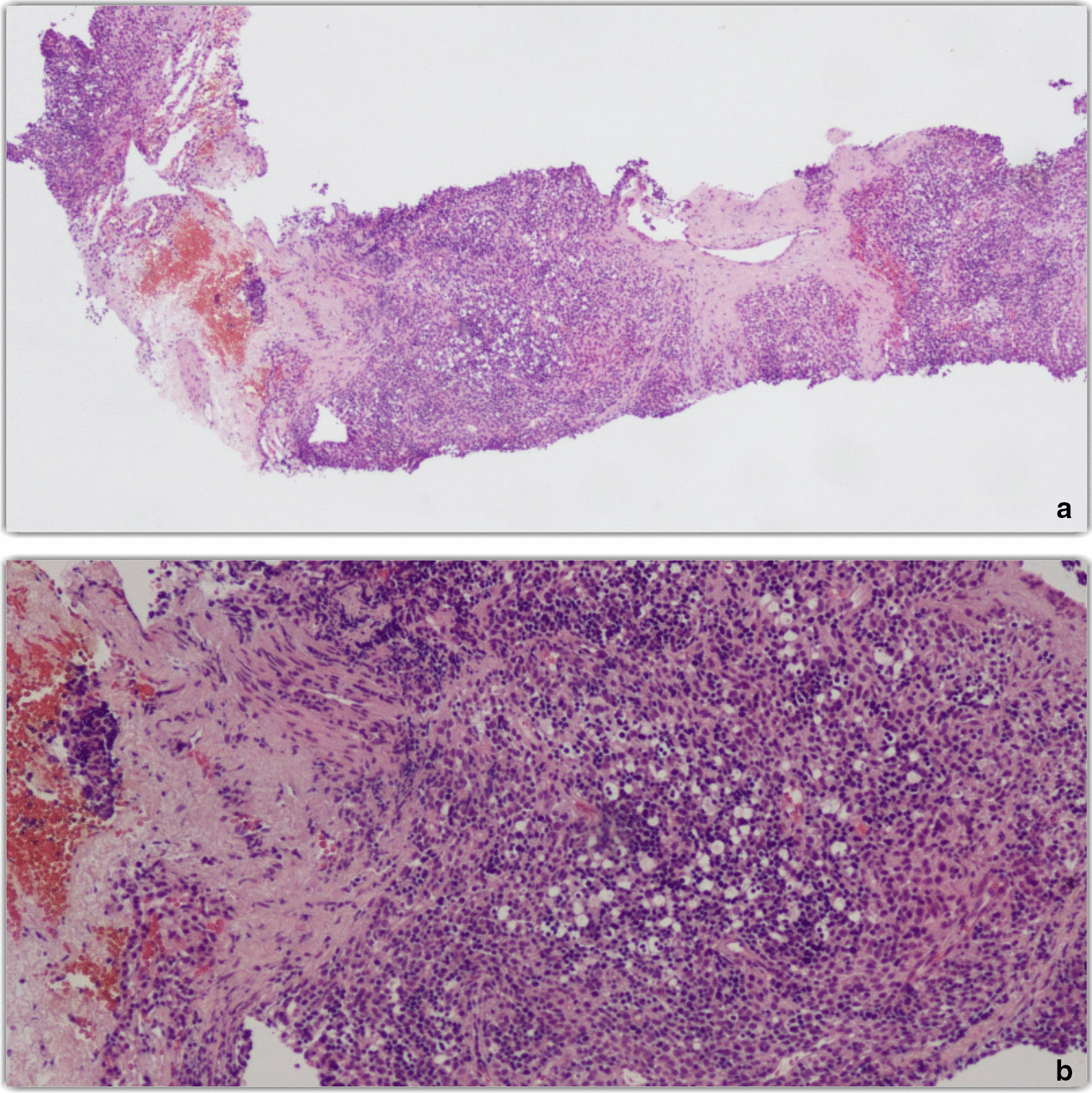
Patient histology. **a** Hematoxylin and eosin (H&E; ×40 view) stained section of thymoma B2 according to WHO classification. **b** Same histological slice in ×100 view

**Fig. 3:**
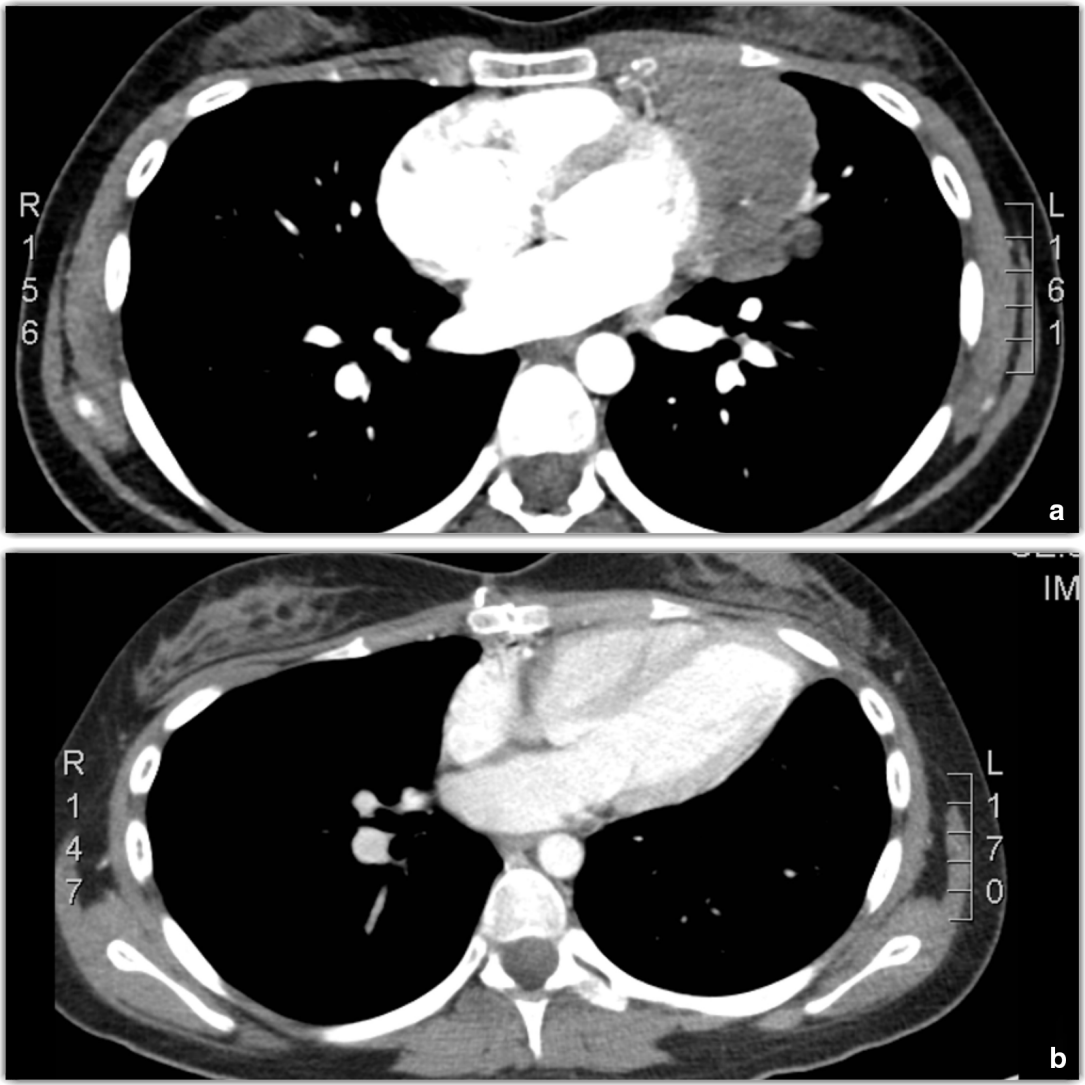
Chest computed tomography (CT) scan at baseline and after surgery. **a** Chest CT scan showing thymoma at baseline. **b** Postsurgical chest CT scan with no residual disease

After completing a second course of CAP chemotherapy with interposed PEX allowing symptom control, radiological assessment in week 6 documented objective partial response according to the Response Evaluation Criteria in Solid Tumors version 1.1 (RECIST 1.1), with 33% tumor shrinkage (maximum diameter 8 versus 12 cm) [20]. This allowed us to perform radical (R0) extended thymectomy, left upper lung lobectomy and subtotal pericardiectomy, with histological diagnosis of Masaoka-Koga stage III type B2 [10, 11]. For patient histology, please see Fig. 2. The patient required early mediastinum and pleural cavity re-exploration due to postsurgical bleeding; persistent hypophonia developed due to postsurgical left vocal cord paralysis. A comparison of CT imaging at baseline and after surgery is shown in Fig. 3. As suggested for Masaoka-Koga stage III, radiotherapy of 50.4 Gy in 28 fractions was given as postoperative treatment in weeks 15–20 [1]. At the last follow-up visit, 6 months after surgery, the patient was in complete remission with gradually improving MGFA class I MG and mild persistent hypophonia [8]. Despite remarkable levels of psychological burden, the patient’s resilience and high-priority treatment goals were fundamental for successful coping.

## Discussion and conclusions

**Fig. 4:**
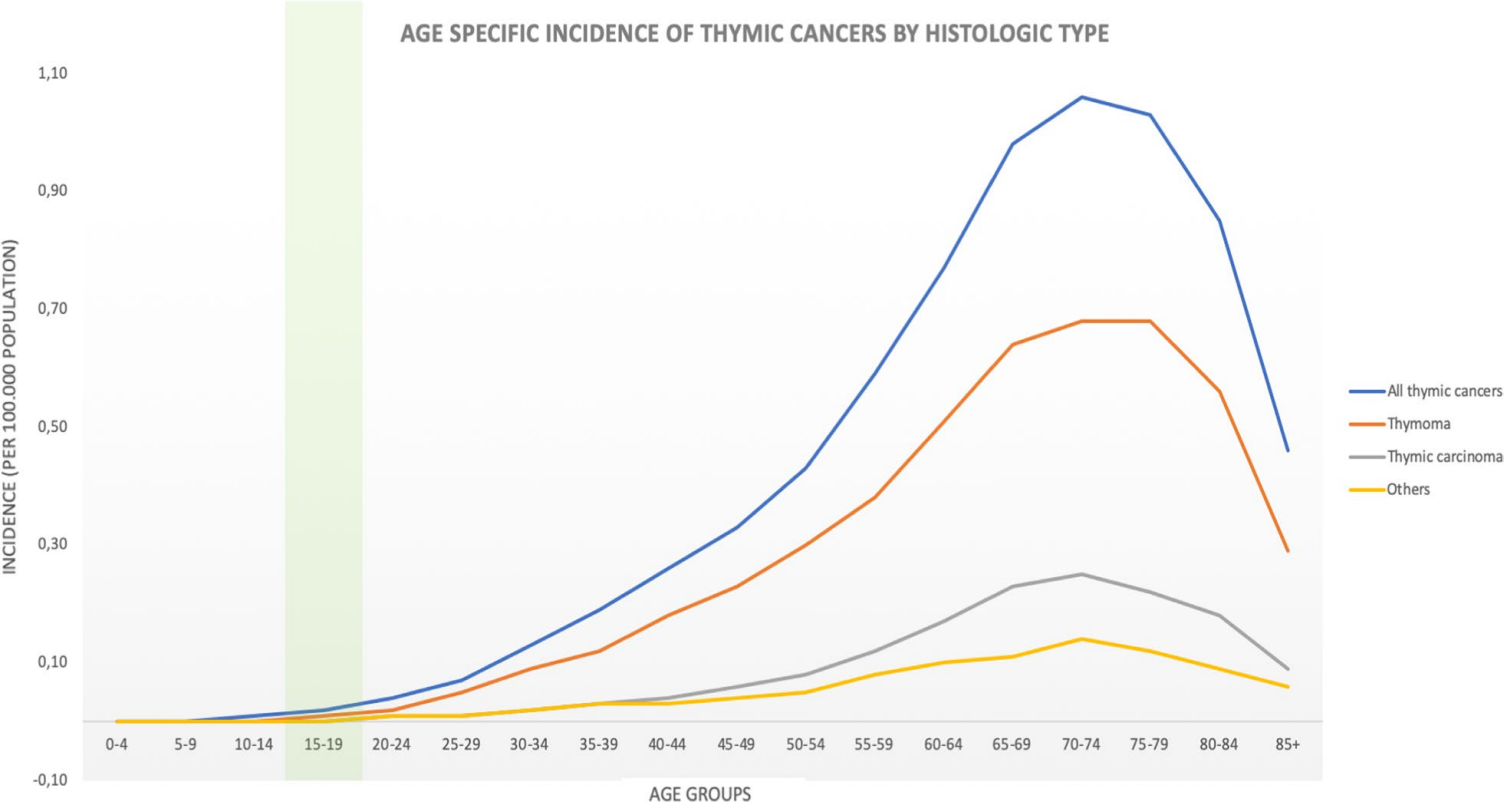
Age-specific incidence of thymic cancer by histological type. SEER*Stat Database: NPCR [National Program of Cancer Registries] and SEER [Surveillance, Epidemiology, and End Results] Incidence—U.S. Cancer Statistics Public Use Database, Nov 2017 submission (2001–2015). The green bar highlights the reported patient age interval (15–19 years)

According to the World Health Organization (WHO), thymoma is the most common variant of thymic epithelial tumors (66.3%), usually presenting with local invasion and seldom as metastatic disease [10, 21]. Overall, thymic epithelial tumors occur at a rate of 0.13–0.32 cases per 100,000 population at risk and are very uncommon in young adults [22, 23]. To our knowledge, the largest retrospective study regarding childhood thymic tumors (< 18 years) in Europe was able to identify only 36 cases during a 12-year period, confirming the extreme rarity of thymic cancer in children and adolescents [24]. Hence, the clinical case reported here has to be considered a peculiar presentation of early-onset thymoma (Fig. 4).

Like thymoma, MG is extremely rare (0.17–2.13 per 100,000 person-years) [25]; however, it affects up to 30% of patients with thymoma [26]. The association of thymoma with MG has been related to the intratumoral generation of mature T cells, although the pathogenic mechanisms remain unclear [27]. MG has been found to be most commonly associated with B thymoma and confers favorable prognosis, since neuromuscular symptoms help in earlier detection of the tumor and thus less advanced stage at diagnosis. Nevertheless, concomitant MG should also be regarded as an adverse risk factor, since it can interfere with cancer treatment, being worsened by many drugs, and it reaches its maximum severity after thymectomy in a proportion of patients [28].

The case reported here, together with those published and summarized in Table 1 [3–6], highlights that chemotherapy is a realistic trigger event of MC in patients with thymoma and MG. The temporary association in all cases indicates a causative relation, and may hamper the continuation of chemotherapy that is a crucial element of multimodal treatment in cases not amenable to upfront radical surgery [1]. No biomarker is available to prove this relation, and clinical findings are crucial for the diagnosis of MC. Other differential diagnoses, such as infusion reaction to cancer chemotherapy, are less likely in the setting of prolonged respiratory failure. Here, a multidisciplinary approach was necessary, and involved neurologists for medical treatment and hematologists for a correct timing schedule of PEX sessions in order to avoid plasma drug displacement. It should be noted that we waited 72 hours from the end of chemotherapy before starting PEX, in order to preserve drug efficacy according to their half-life.

**Table 1.** Comparison between present case and 4 other published cases

	Fujiwara *et al.* [3]	Qureshi *et al.* [4]	Solak *et al.* [5]	Ng *et al.* (6)	Present case report
Sex	Female	Male	Male	Male	Female
Age (year)	44	24	45	49	18
Histology	B1 Thymoma (WHO)	Thymoma	Thymoma	NE	B2 thymoma (WHO)
Staging	IVA (Masaoka-Koga)	III (Masaoka-Koga)	Advanced unresectable	Advanced unresectable	III (Masaoka-Koga)
AchR Ab level (nmol/l)	13	NE	74	NE	43
Chemotherapy regimen	Primary CAMP	Postoperative cisplatin and doxorubicin	Single-agent cisplatin	Doxorubicin, cisplatin, etoposide	Primary CAP
Myasthenic crisis onset time	During chemotherapy	8 hours after chemotherapy	1 week after chemotherapy	A few hours after chemotherapy	During chemotherapy
Tracheal intubation	No	Yes	Yes	Yes	Yes
Plasma exchange	No	Yes	Yes	Yes	Yes
Chemotherapy continuation in ICU	No	No	No	No	Yes
Patient outcome after myasthenic crisis	Alive	Death	Alive	Alive	Alive

*AchR* Ab acetylcholine receptor antibodies, *CAMP* cisplatin, doxorubicin, methylprednisolone, *CAP* cyclophosphamide, doxorubicin, cisplatin, *ICU* intensive care unit, *NE* not evaluable, *WHO* World Health Organization

To our knowledge, this is the first reported case of chemotherapy continuation despite the need for mechanical ventilation due to a chemotherapy-induced MC. Radical treatment was dependent on primary chemotherapy, as upfront surgery had already been ruled out. Besides, we could not confidently assume that, after MC resolution, the patient would have been able to receive further chemotherapy due to aggravation of symptoms. We conclude that special attention is warranted in patients with thymoma and MG with regard to the development of MC. Ideally, optimal control of myasthenic symptoms should be achieved before initiation of anticancer treatment. Nonetheless, chemotherapy-induced MC may complicate anticancer therapy administration, according to this evidence. We recommend that patients with thymoma and MG be hospitalized in tertiary medical centers for chemotherapy and that outpatient management be avoided. The lesson learned from the present case is that, in selected cases of advanced thymoma, paradoxical worsening of MG during chemotherapy should not be considered an absolute contraindication for continuation of primary chemotherapy with curative intent.

## Abbreviations

CAP: Cisplatin, doxorubicin and cyclophosphamide
CT: Computed tomography
CTCAE v5.0: Common Terminology Criteria for Adverse Events version 5.0
Gy: Gray
ICU: Intensive Care Unit
MC: Myasthenic crisis
MG: Myasthenia gravis
MGFA: Myasthenia Gravis Foundation of America
^18^PET-FDG: 18-Fluorodeoxyglucose positron emission tomography
PEX: Plasma exchange
RECIST: Response Evaluation Criteria in Solid Tumours
WHO: World Health Organization
